# An Interactive Vision‐Based 3D Augmented Reality System for In‐Home Physical Rehabilitation: A Qualitative Inquiry to Inform System Development

**DOI:** 10.1111/hex.70020

**Published:** 2024-10-23

**Authors:** Afolasade Fakolade, Adriana C. Salvia, Siona Phadke, Manuela Kunz

**Affiliations:** ^1^ School of Rehabilitation Therapy Queen's University Kingston Ontario Canada; ^2^ Providence Care Hospital Kingston Ontario Canada; ^3^ Digital Technologies Research Center, National Research Council Canada Ottawa Ontario Canada

**Keywords:** augmented reality, immersive systems, older adults, physical rehabilitation, total joint replacement

## Abstract

**Background:**

Postoperative physical rehabilitation is crucial after total joint replacement (TJR). However, completing the recommended levels of postoperative physical exercise is challenging for many older adults with TJR. Lack of adequate postoperative physical exercise has negative consequences on rehabilitation outcomes. Innovative rehabilitation tools for postoperative physical exercises are needed to ensure successful rehabilitation outcomes among older adults with TJR.

**Objective:**

The aim of this study is to explore key knowledge users' perspectives about how to design an interactive vision‐based three‐dimensional augmented reality system (3D ARS) to support in‐home postoperative physical rehabilitation for older adults with TJR.

**Methods:**

We conducted a qualitative descriptive study involving 11 semi‐structured interviews and six focus groups with 42 older adults with TJR and four unrelated family caregivers. Data were analysed using thematic analysis.

**Results:**

Participant insights were grouped into two main themes: (1) dreaming up possibilities and (2) being pragmatic. The first theme captured participants' reflections on the potential utility of a 3D ARS for postoperative physical rehabilitation and features that could be embedded in the 3D ARS to support successful postoperative physical rehabilitation. The second theme captured participants' reflections on practical issues and considerations that could impact access and usage of the 3D ARS.

**Conclusion:**

These findings provide researchers, rehabilitation providers and system developers with the foundations for designing, implementing and evaluating innovative augmented reality tools that support effective in‐home physical rehabilitation among older adults with TJR.

**Patient or Public Contribution:**

Research users (i.e., individuals and organisations invested in and using the research findings) were actively engaged throughout this work. Specifically, a meeting was held between the research team and representatives of an Expert by Experience team (individuals with lived experience), which was established to support the National Research Council's (organisation) Aging in Place programme. During this meeting, the idea to develop and evaluate an ARS for postoperative physical rehabilitation of older adults with TJR was supported. Research users had the opportunity to review the current study protocol and provide feedback on the study design, offering direction to maximize the relevance and usefulness of our findings to the National Research Council Canada's Aging in Place programme. Research users contributed to participant recruitment efforts and the development of the interview guide. Two Experts by Experience also agreed to be on the Advisory Panel for this multi‐phased study, supporting active engagement and centring the voice of research users in knowledge creation and implementation. These experts reviewed a brief report of the current study findings, and continue to guide how the study findings are used to inform the next phase of this multi‐phased research.

## 1 Introduction

Total joint replacement (TJR) is recommended for alleviating pain and restoring function in older adults with moderate to severe osteoarthritis when conservative first‐line management options are no longer effective [1, 2]. Postoperative physical rehabilitation, including supervised (clinic‐based) and unsupervised (in‐home) exercises, improves outcomes such as pain, mobility and functional independence among older adults after TJR [3, 4, 5]. However, completing recommended levels of postoperative physical rehabilitation exercises, particularly in the in‐home recovery phase, is challenging for many older adults with TJR [6]. Lack of adequate exercise often results in suboptimal rehabilitation outcomes [7, 8, 9, 10]. Supporting older adults with TJR in completing recommended in‐home postoperative physical rehabilitation exercises requires innovative approaches to ensure positive rehabilitation outcomes.

Immersive systems, such as virtual reality (VR) and augmented reality (AR), offer tremendous potential for in‐home postoperative physical rehabilitation of older adults with TJR [3, 11, 12]. Indeed, advances in and availability of technology tools, coupled with the surge in virtual healthcare during the COVID‐19 pandemic, have created an impetus for patients and healthcare providers to use these options as an alternative, enhancement or complement to traditional face‐to‐face physical rehabilitation services in hospitals or outpatient clinics [12, 13].

VR systems immerse the user within an entirely simulated space while blocking out visual stimuli from the natural world [14]. On the other hand, AR systems allow users to see and interact with the natural world overlaid with virtual three‐dimensional images, ‘augmenting’ the user's visual field with the information necessary to perform a current task [14]. AR systems allow knowledge gained in a therapeutic setting to be used in daily life by providing an interactive experience anchored in physical reality while ensuring that the user is aware of and can respond to potential environmental dangers [15]. Although VR systems rely primarily on head‐mounted displays, AR systems can be implemented using various technologies, including head‐mounted displays, conventional monitors or TVs. VR systems also have well‐known adverse side effects, including VR or cyber sickness. AR systems, especially when using monitors or TVs, are less prone to these adverse side effects [16].

Although the interest in immersive systems for in‐home physical rehabilitation is growing [3, 17, 18, 19], attempts to generate evidence in this area have focused primarily on VR systems, with less attention paid to leveraging the potential of AR systems [3, 20, 21, 22]. The limited studies exploring the utility of AR systems for physical rehabilitation tend to focus on individuals with neurological deficits [23, 24, 25, 26] or on enhancing system design and usability [27, 28]. We know very little about how AR systems can support in‐home physical rehabilitation for adults with TJR, nor about their AR system requirements and preferences.

End‐user input in all stages, from initial identification of need to actual development and testing of technology‐supported systems, is essential to ensure relevance, identification of outcomes aligned with users' needs and successful implementation [29, 30]. Actively engaging end users in the creation process can also prevent tokenistic inclusion, wherein end users are asked to validate the system after creation rather than centring their voices in the creation process [31, 32]. Without gathering information on end users' requirements and preferences, researchers risk wasting minimally available resources to develop systems that are not relevant or tailored to the needs of the end users [33].

A range of participatory approaches and frameworks can support this user‐engagement process. One such approach involves using an anticipatory method, commonly used in the policy development context, to explore views and attitudes towards emerging technologies [34]. This approach involves collecting data from participants with limited to no experience with the proposed technology to reduce technological risk and inform whether further research and development should occur in the proposed area [34]. This approach has been used in stroke rehabilitation to successfully explore the perspectives of stroke survivors and health professionals about how AR systems could be used to support inpatient stroke rehabilitation [35]. This current study was positioned within an anticipatory approach and aimed to explore end users' perspectives about how to design an interactive vision‐based three‐dimensional augmented reality system (3D ARS) to support in‐home postoperative physical rehabilitation for older adults with TJR. This study is part of a larger multi‐phased, multi‐methods project to develop, evaluate and implement an interactive vision‐based 3D ARS for in‐home postoperative physical rehabilitation after TJR.

## 2 Methods

This study used a qualitative descriptive design [36] to capture the perceptions of end users and explore how the AR 3DS can be designed to address the needs of older adults with TJR. This design utilizes a naturalistic perspective to discover straightforward descriptions of a phenomenon [37]. A qualitative descriptive approach does not go into a deep exploration of interpretation but keeps the researcher close to the data [36]. It allows the researcher to discover perspectives and understandings of a phenomenon [38], compared to a phenomenological approach, which explores lived experiences [39]. A qualitative descriptive approach is suitable for areas where little is known about the topic under investigation [40], as in this study. Further, this approach allows data to be translated to inform the development of resources and tools tailored to the needs of the target groups [41, 42].

### 2.1 Participant Recruitment

We recruited older adults with TJR and their family caregivers using study flyers distributed through community organisations providing support services for older adults with osteoarthritis (e.g., Arthritis Canada), the physical rehabilitation department of a local hospital, libraries, seniors centres and outpatient rehabilitation clinics. We also used a snowballing technique, where participants were asked to share the study flyer with peers or colleagues interested in participating. A higher number of older adults with TJR (vs. family caregivers) were targeted for recruitment, as they are the potential end users of the proposed 3D ARS. Study flyers contained a brief overview of the study, requirements for participation, information about compensation of a $20 gift card to be sent to participants' email addresses after study participation and the research office email address. Prospective participants were invited to email the research office to express their interest in participating in the study. A research assistant was available via email to answer any questions about the study. Upon contact, the research assistant emailed them a PDF copy of a detailed information letter. Participants were invited to review the information letter and confirm eligibility to participate in the study through an online survey via Qualtrics (Qualtrics, Provo, Utah, USA).

### 2.2 Participant Eligibility Criteria

Eligibility criteria included (1) being an older adult (≥ 65 years old) who had undergone TJR ≥ 6 months ago, allowing us to engage those with some postoperative physical rehabilitation experiences, or (2) being an adult (≥ 18 years old) family caregiver for an older adult with TJR. Additionally, all participants needed access to an internet‐enabled computer, tablet or smartphone to participate in the study. Participants were not required to have previous experience using either AR or VR technology.

### 2.3 Data Collection

Data collection was online, wherein participants who indicated consent, confirmed eligibility and provided demographic information in an online survey were asked to indicate their preference to complete one individual interview or attend one focus group via Zoom (Zoom Video Communications, Inc., San Jose, CA, USA). We offered both options to reduce participation barriers and ensure consideration of participants' preferences and scheduling availability [43]. Once participants completed the online survey, they were contacted by a research assistant to schedule the day and time for an individual interview or a focus group, depending on their preferred selection. During the scheduling call, the research assistant also asked and recorded whether participants required accommodations or training to participate in an interview or a focus group via Zoom. No participants requested accommodations or training.

We asked the same questions, informed by the Technology Acceptance Model [44], during both individual interviews and focus groups—File S1. Interviews and focus groups were conducted in parallel. Each interview and focus group began with a definition of AR. To promote conversation about the proposed 3D ARS, the interviewer shared a PowerPoint slide deck using the share screen function on Zoom. The slide deck included preliminary design details about the proposed 3D ARS, including superimposed computer‐generated images of an individual performing physical exercises. Participants were informed that the proposed system would have a small, integrated 3D camera—File S2—to observe individuals performing their recommended postoperative exercises and play the camera feed in parallel on a smart TV screen (similar to a mirror). Additional information (e.g., prompts for postural alignment and joint range‐of‐motion angles) may be superimposed onto this mirror image to guide individuals to perform their exercises correctly and for the best outcomes.

Data collection was a dynamic process, whereby preliminary findings from the first three individual interviews and the first focus group were incorporated into subsequent ones, enabling regular appraisal of information power [45]. For example, preliminary analysis of the transcripts indicated that some participants in the earlier interviews and focus group found it challenging to conceptualise the proposed 3D ARS and answer questions that asked them to imagine an ideal system that would support and guide their movements during exercises. Therefore, we revised our PowerPoint slide deck by adding an image of the proposed integrated 3D camera. We also added several more computer‐generated images and examples of different types of AR displays, avatars, virtual environments and options for system navigation to stimulate conversation about features that could be incorporated into the system.

Data collection was discontinued when the team agreed that the data had adequate power to address the study aims. Digital recordings were professionally transcribed (intelligent verbatim) by a transcription company.

### 2.4 Positionality

This study is framed within ontological relativism and epistemological subjectivism, assuming the existence of multiple, subjective and mind‐ and context‐dependent realities. We acknowledge that knowledge is co‐constructed through interactions among researchers, participants and the broader sociocultural environment [46]. The first author and a research assistant conducted the individual interviews and focus groups. These researchers have over 10 years of combined experience conducting qualitative research involving interviews and focus groups. In addition to these researchers, the 3D ARS development team representative also attended several focus groups. The research team has combined multidisciplinary clinical and research experiences in physiotherapy, computer science and biomedical engineering, psychology, ageing and qualitative research. None of the researchers had a pre‐existing relationship with the participants.

### 2.5 Data Analysis

Descriptive statistics were computed for the sample using SPSS version 28.0 (IBM SPSS Statistics, Armonk, NY). Qualitative data were analysed using thematic analysis [47]. The second author or a research assistant removed any identifying information and checked the accuracy of the transcription. Transcripts were imported into NVivo (Version 14, QSR International Pty Ltd, Melbourne, Australia) to facilitate efficient coding and data organisation. The first step in the analysis involved a period of data familiarisation, which involved the second and third authors repeatedly reading the first three transcripts upon receipt from the transcription company. Then, they systematically generated codes by identifying key points of interest within the data. The first and last authors acted as ‘critical friends’ to challenge initial codes, contribute to developing new codes and stimulate reflection on alternate perspectives [48]. A coding frame, which included the codes and their conceptual descriptions, was subsequently developed and refined in a research team meeting with all the authors—File S3. Then, the second and third authors proceeded to code subsequent transcripts on an ongoing basis as they were received. Any new codes identified were discussed in weekly team meetings with the first author and included in the coding frame, as appropriate. The initial three transcripts were then recoded by the second and third authors. Once all the transcripts were coded, the second and third authors collapsed extracted codes with shared meanings into potential themes. All authors reviewed the themes and engaged in ongoing discussions to clarify each theme's scope, boundaries and alignment with research objectives. Finally, the authors created a coherent written report, incorporating exemplar quotations to enhance understanding of the themes. The draft report was shared with representatives of the Experts by Experience team during a project meeting with the first and last authors, and with our Advisory Panel for feedback.

### 2.6 Rigour and Trustworthiness

Consistent with our philosophical worldview, we chose four criteria for rigour: resonance, meaningful coherence, ethical conduct and reflexivity. *Resonance* was ensured by offering thick descriptions and interpretations, enhancing transferability to different situations (naturalistic generalisations). We used direct quotations from participants to allow readers to independently assess our interpretations and the appropriateness of our themes. *Meaningful coherence* was achieved using methods and procedures that fit the study's goals. *Ethical conduct* was maintained through informed consent and participant anonymity. *Reflexivity* was ensured using ‘critical friends’ to stimulate reflection and explore alternative explanations and interpretations. These strategies are consistent with contemporary approaches for rigour in qualitative research [48].

## 3 Results

Forty‐six participants (42 older adults with TJR and four caregivers) were enrolled into the study and provided demographic and background information—see Table 1. Eleven individual interviews (each lasting 45–60 min) and six focus groups (each lasting 60–90 min with four to six participants in each group) were conducted. We identified two main themes from the analysis of interview and focus group transcripts: (1) *dreaming up possibilities* and (2) *being pragmatic*. Below, we present the themes in detail, including exemplar quotations for context. Quotations are referenced by participant identifiers in brackets. For instance, OA01 denotes an older adult, CG01 denotes a family caregiver, FG01 denotes focus group 01 and ISI01 denotes individual semi‐structured interview 01.

**Table 1:** Characteristics of enrolled participants (*n* = 46) who completed the background survey.

Variable	
Age (years), mean (SD)	70.1 (10.1)
Years since joint replacement, mean (SD)	5.48 (6.9)
Type of knowledge user	
Older adult	42 (91.3)
Family caregiver	4 (8.7)
Gender, *n* (%)	
Male	11 (23.9)
Female	29 (63.0)
Transgender man	1 (2.2)
Not reported	5 (10.9)
Province of residence	
Alberta	3 (6.5)
British Columbia	11 (23.9)
Manitoba	1 (2.2)
Ontario	28 (60.9)
Prince Edward Island	1 (2.2)
Quebec	1 (2.2)
Not reported	1 (2.2)
Highest education obtained	
Technical or Trade School	2 (4.3)
High school/GED	7 (15.2)
College/CEGEP	9 (19.6)
Bachelor's degree	13 (28.3)
Master's degree	8 (17.4)
Doctoral degree	2 (4.3)
Not reported	5 (10.9)
Family income	
< $20,000	2 (4.3)
$20,000–29,999	3 (6.5)
$30,000–59,999	11 (23.9)
$60,000–89,999	9 (19.6)
$90,000–129,999	2 (4.3)
$130,000 or more	10 (21.7)
Rather not disclose	4 (8.7)
Not reported	5 (10.9)
Type of joint replacement	
Right Hip	8 (17.4)
Left Hip	2 (4.3)
Right Knee	2 (4.3)
Left Knee	6 (13.0)
Multiple lower limb joint replacements	17 (37.0)
Multiple lower limb joint replacements and bilateral shoulder joint replacements	3 (6.5)
Not reported	8 (17.4)

### 3.1 Theme 1: Dreaming up Possibilities

This theme included two sub‐themes that captured participants' reflections on the potential utility of a 3D ARS for postoperative physical rehabilitation and beneficial features that could be embedded in the proposed 3D ARS to support postoperative physical rehabilitation.

#### 3.1.1 Subtheme 1: Potential Utility of a 3D ARS for Postoperative Physical Rehabilitation

Participants displayed high support and enthusiasm for the proposed interactive vision‐based 3D ARS. Many participants shared that they were unprepared and lacked the tools to deal with the numerous challenges that they experienced on the long TJR recovery journey, including a lack of motivation to complete the necessary but often painful postoperative exercises. They also shared challenges with access to and availability of rehabilitation professionals to guide and provide feedback on home exercises after hospital discharge. An older adult participant in FG01 commented:

I had never had surgery before, other than having my tonsils out. I had no idea what to expect in terms of pain. No idea what to expect in terms of short‐, medium‐ and long‐term recovery.

(OA5; FG01)

All participants agreed that an interactive vision‐based 3D ARS could help address these gaps. Specifically, they perceived that the system could be used as a tool to support their continuous engagement in the recommended exercises, tracking progress and providing real‐time feedback and guidance (e.g., prompts displayed on the screen for proper posture and joint alignment, flashing green lights or celebratory starbursts for achieving goals) during postoperative exercises. Another older adult participant and an unrelated family caregiver, respectively, shared this reflection in FG01:

I think—like to me, the main benefit would be immediate feedback. Like if I'm doing the exercise incorrectly or I'm going—whatever—if I could do it in a better manner and I got immediate feedback, that would seem to be the main benefit.

(OA09; FG01)

I think if it had a weekly recap of her progress, I think that would be beneficial. So, if she can see herself how much progress she's made, I think that would motivate her to continue with the exercises. And I think that it, being an interactive program, I think that already helps because it's interactive. So, it's not as boring as looking at a piece of paper.

(CG01; FG01)

Some participants, particularly those who had experienced challenges accessing services, including educational resources and support groups pre‐ and postsurgery, further shared that they saw the potential for the 3D ARS to be used as a tool to build and/or increase their social connectivity. These participants perceived that promoting social connectivity through the 3D ARS could foster the sharing of experiences with others in similar situations and vicarious learning. An older adult participant shared the following reflection during their individual interview:

If there was a way that you did that [exercise] weekly with people that were at a similar stage of—because it's really only the first couple of months where you are like at your wits' end, right? Where you're in a lot of pain, you're on dope; you're like barely moving, really, right? So, especially during that time, it is really important to keep moving, and it's really important to reach out and get support, right? So, if there was a way that yes, you could meet with people and check in and be encouraged by what they're doing, right, that's good.

(OA06; ISI01)

#### 3.1.2 Subtheme 2: Beneficial Features That Could Be Embedded in the Proposed 3D ARS to Support Postoperative Physical Rehabilitation

Participants shared that they lacked the knowledge to identify indicators of postoperative complications during the recovery phase. They discussed and recommended including sensors to track and alert users and their healthcare providers to issues indicative of potential postoperative complications, such as a progressive reduction in range of motion, swelling at the affected joint or movement abnormalities during exercises. For instance, an older adult offered this recommendation during FG04:

The sensors, you can strap on your leg or whatever body part you need to move. That would be good. And also … like a camera, you could attach to your TV so you could be watching, but then also, they [healthcare providers in circle of care]. And someone somewhere would be providing feedback on what you're doing, if you're doing it accurately.

(OA23; FG04)

A family caregiver further shared this reflection during their individual interview:

I think for her, though, that's something that if whether it be the goggles, or an experience that it was on the TV, where they [healthcare providers in circle of care] were able to speak to her using sensors, that type of thing, where there was that type of biofeedback between her and a screen, whether that's through goggles, TV, however it is.

(CG03; ISI02)

Another suggestion was to include features that allow users to customize the 3D ARS experience by providing various types of exercises, virtual backgrounds and imagery, music matched to the individual's age and system activation options. Participants perceived that a customisable experience would also motivate continued use of the system, stating that it would be ‘An improvement to the current practice of receiving a piece of paper with some exercise drawings’ (OA18; FG04).

We noted that participants across both individual interviews and focus groups had two distinct perspectives about the system activation options. Although some participants considered voice activation features and virtual avatars as exciting features for ease of use of the system, others expressed caution and described frustration with the previous use of such features. Remote control was suggested as an alternative for activating the system, with many participants stating that they were more familiar with this option. Others recommended a real‐life image of a person rather than virtual avatars. For instance, an older adult in FG04 shared this perspective:

Oh, wouldn't voice activation be great? Although sometimes voice activation, like when I said my grandson taught me how to send texts using voice activations, and I've said really rude things to people through that voice activation on [name of device], right? So, I mean, if it worked, if not, a remote control would be easier. Again, I would say that for the widest breadth of people, and this is my presumption, I think the remote control might be the best because we're all more familiar with that.

(OA25; FG04)

Another older adult participant in FG06 commented:

I don't want a model, I don't want a university, high school body. Just a real person 60+ who I feel comfortable with, who's doing the movements. I don't like the avatar. They're not part of my generation for me, don't work.

(OA40; FG06)

### 3.2 Theme 2: Being Pragmatic

This theme captured participants' reflections on practical issues that could impact system access and usage. Participants discussed considerations related to the cost of the system, the level of technological proficiency required to use the system and the physical space needed for system set‐up. Several participants shared that as the primary patient population for TJR are older adults who are often on a fixed/definite income, asking them to purchase a ‘fancy’ 3D ARS and other related equipment (e.g., smart TV) may be a cost‐prohibitive financial investment. Participants suggested that given that TJR recovery is time‐limited, the 3D ARS should be offered free of charge or for renting at discounted costs through their healthcare provider or community‐based settings rather than purchasing. For instance, two older adults shared these reflections during a focus group and an individual interview:

Because let's face it, most of the people who are getting their joints replaced tend to be elderly, and the last thing you want to do when you're retired or nearing retirement is to fork out extra money unless it's going to really help you, you know? You've got to be convinced that it's going to be worth it.

(OA42; FG06)

I think that would be a really good option, again, especially in more remote communities where the people don't have the option of a physiotherapist. Certainly, the technology, if it could be rented [for renting at discounted costs], would be ideal.

(OA37; ISI11)

Others cautioned against the proposed smart TV‐only option and recommended system compatibility with desktops, laptops or tablets, stating that many older adults already have these tools and would find it cheaper and easier to integrate with the proposed 3D ARS. An older adult shared this reflection during their individual interview:

I know lots of people that don't have a TV at all. So just like if you want to make it accessible, it may need to connect to a laptop or a tablet or something.

(OA06; ISI01)

A related conversation concerned technological proficiency, focusing mainly on understanding how users would set up the system's integrated 3D camera to communicate with and project images onto a smart TV. When informed that a user would plug in the camera to communicate directly with a smart TV, some participants shared that it might be challenging for some older adults to complete the system set‐up independently. Others wondered how older adults would be able to interpret the information from the integrated 3D camera displayed on the smart TV screen. Participants recommended minimal but clear information and details on the screen (e.g., joint angles superimposed on a virtual body representation). Another recommendation was to provide additional 24/7 technical support for learning how to use the system and troubleshooting any issues with the system's operability. For instance, an older adult offered this recommendation during FG06:

My suggestion is since most people who will be doing this are older and may or may not be computer literate, or even if they are, they're not necessarily good with, you know, smart TVs, you have to have software that is very, very simple and self‐correcting. You know? When they say it's just not working, which you'll hear all the time – you have to be able to go in from your side, diagnose what they've done, and reset the system. Because they will log themselves out every time. Right? The best way is to make it so there's very little interaction to set it up and run. It should just run itself as much as it can absolutely idiot‐proof.

(OA44; FG06)

Finally, some participants voiced concerns about the system's physical space requirements. Specifically, participants were informed that preliminary modelling work completed by the 3D ARS development team had indicated that the system would require a 2‐m functional range to capture all joint angles and body movements optimally. They wondered whether older adults, who had often downsized to smaller residences, would have sufficient space to accommodate the 3D ARS. An older adult in FG06 commented:

That sounds like that would require a lot more room, maybe, than just standing in one place doing these exercises. I don't have at the moment in the house a room that has a lot of floor space where I could get down and actually feel like even the yoga that I'm doing is … I've got this little piece of a mat, you know, that I stretched out in one room.

(OA45; FG06)

## 4 Discussion

This study is nested in a larger, multi‐phased, multi‐methods research programme focused on developing, evaluating and implementing an interactive vision‐based 3D ARS for older adults after TJR. To inform the system's development, we explored end users' perspectives on designing the system to address the in‐home postoperative physical rehabilitation needs of older adults with TJR. The use of AR systems in TJR rehabilitation is an emerging concept [3, 17]. Therefore, this study's anticipatory approach supported participants in imagining a future of postoperative TJR rehabilitation with AR systems and centring their voices in the knowledge‐creation process [31, 32].

We identified two major themes from our analysis. The first theme identified was *dreaming up possibilities*, which included two subthemes: *the potential utility of a 3D ARS for postoperative physical rehabilitation* and *beneficial features that could be embedded in the proposed 3D ARS to support postoperative physical rehabilitation*. The second theme identified was *being pragmatic,* which captured participants' reflections on practical issues and considerations that could impact system access and usage. Herein, we situate our *key* findings amidst published literature on immersive technologies in rehabilitation.

Overall, participants indicated their support and enthusiasm for the proposed interactive vision‐based 3D ARS. This finding is important to highlight, as patient compliance with immersive technology‐supported rehabilitation and intervention outcomes can be affected by the perception of the technology [23, 49]. Contrary to our findings, older adults are often portrayed as resisting technological advances, having low enthusiasm and support for new technology, and demonstrating a lack of comfort using these systems [50]. Although the level of support and enthusiasm displayed by our participants bodes well for the future implementation of the proposed 3D ARS, it may also reflect the current healthcare climate following the COVID‐19 pandemic, where the role of technology in rehabilitation has gained significant importance [51].

Participants perceived that the 3D ARS might help promote continuous engagement in recommended levels of unsupervised postoperative physical exercises at home after hospital discharge. We know that inpatient rehabilitation does not deliver superior outcomes compared with home‐based rehabilitation after knee arthroplasty [52] and that many older adults prefer home‐based exercises but often lack appropriate tools to support engagement [53, 54]. Thus, developing the proposed 3D ARS is a timely and relevant endeavour that offers an alternative model of service delivery to meet the in‐home physical exercise needs and preferences of older adults with TJR.

A desire for a system that could provide real‐time feedback and guidance during postoperative exercises and track exercise‐related progress was expressed by both older adults with TJR and family caregivers. This finding is not surprising, given that real‐time monitoring of patients during in‐home exercise regimens is a well‐recognized rehabilitation challenge [23]. Our findings suggest that an immersive system capable of displaying assistive health parameters during exercises, while continuously analysing and updating user information, can improve exercise adherence and help prevent pitfalls during unsupervised remote exercise training [12]. Other researchers have shown that immersive systems that offer synchronous and asynchronous monitoring and provide performance‐related real‐time feedback on a user's activity can help track correct movements and retrain patients' movement patterns in stroke rehabilitation [55].

Additionally, participants reflected on the potential for an interactive 3D ARS to facilitate social connectivity and sharing experiences with other older adults with TJR. Social isolation continues to be a significant issue for older adults with TJR, particularly during the recovery phase [56]. In their position paper, Brox et al. [57] call for system developers and researchers to consider social interactions in the context of immersive systems. More recently, researchers have reported that immersive systems continue to have an untapped potential to improve social relationships, well‐being and quality of life among older adults [50]. We extend these findings by highlighting a strong desire for social interaction in the context of AR‐supported home‐based physical rehabilitation after TJR. Including online multi‐user modes or simulated groups in the proposed 3D ARS may contribute to social interaction during postoperative exercises and thus increase the adherence of users to their recommended exercise regimens.

Our participants identified beneficial features that could support postoperative physical rehabilitation, including sensors to monitor and flag potential complications, information‐sharing capacity and customisation of options. Recent AR rehabilitation research [58, 59, 60] has documented the benefits of incorporating wearable sensors for data gathering, including dynamic and kinematic information associated with upper and lower extremity motion. Sharing information from these sensors with healthcare providers can alert them proactively about possible issues and avert complications, ultimately improving postsurgical rehabilitation outcomes [61].

A previous study exploring the merits of personalized versus standard scenarios in immersive system‐supported rehabilitation indicated that a lack of customisation could, over time, result in diminished user engagement and motivation [62]. As we develop the 3D ARS, we must consider how we can incorporate customisable options to ensure that older adults with TJR have choice and control over how their postoperative rehabilitation needs are addressed. In addition to providing options for virtual backgrounds and imagery, we may also explore including a feature (e.g., a secured video call) allowing users access to a rehabilitation therapist for timely and ongoing customisation of exercises as necessary.

Similar to previous research [35, 53], our findings indicate that the ideal 3D ARS for postoperative TJR rehabilitation should be offered free of charge [or at discounted costs for renting] and easy to set up and use. Participants' perspectives regarding potential system costs draw attention to issues of digital equity and the importance of efforts to address the existing divide in access to digital infrastructure that gives some individuals undue advantages over others [63, 64]. A focus on digital equity during system development and implementation will ensure that the 3D ARS and its supporting hardware remain accessible to all older adults, not just those with financial and geographical resources, and will ultimately contribute to efforts to reduce the social and economic exclusion of some older adults with TJR. For our participants, ease of use implied simple instructions to access the proposed system and that the technology should ‘just work’. Our participants' expectations are not unlike the general expectations for the reliability of other technologies, such as computers and cell phones. Previous research has shown that screen freezes, problems with movement tracking sensors and the need for ongoing system repair are cumbersome and can impact the adoption and use of immersive systems [61, 65]. Our findings indicate that older adults with TJR and family caregivers desire a means of troubleshooting technological issues with the proposed 3D ARS to ensure ease of use. Here again, a secured video call feature may allow users access to a therapist or system engineer for troubleshooting system issues in a timely fashion when necessary.

Finally, perspectives on the physical space considerations expressed by our participants suggest that an initial site visit to the user's home to evaluate the adequacy of space and furniture for interacting with the system [53] may be needed as part of the training provided for users. Alternatively, options for the proposed 3D ARS to guide the user through an intuitive and flexible set‐up procedure could be explored to keep system development costs low.

## 5 Strengths and Limitations

A strength of this work is the inclusion of a sample comprised mostly of older adults with TJR (91%) who are the potential end users of the proposed 3D ARS. Also, a representative of the 3D ARS development team was included in some of the focus groups and the data analysis process. This, in addition to the research team's combined multidisciplinary clinical and research expertise, facilitated links across diverse fields and enhanced nuanced interpretations of the data. Nevertheless, several limitations warrant attention. First, participants' views may not fully represent the general perspectives of older adults with TJR, as individuals who declined participation may hold different opinions. Second, we must acknowledge the inherent bias introduced when most participants were highly educated. It is possible that participants' higher education facilitated their ability to engage in this future‐focused idea generation for a proposed 3D ARS. Finally, our participants did not have experience with the 3D ARS, and although this may be considered a limitation, it is representative of the general population.

## 6 Conclusion

An interactive vision‐based 3D ARS to support in‐home physical rehabilitation of older Canadians after TJR has the potential to dramatically transform rehabilitation delivery to meet the target population's exercise needs and preferences. This study provides a preliminary list of user‐informed recommendations to support system design and development. As with key knowledge user consultations in general, the recommendations provided by participants are not meant to be exhaustive but rather illustrative of key potential opportunities and ideas for system development. The next step in this research programme is to develop a consensus on the design features in a minimum viable 3D ARS prototype. This work will enable us to deliver a prototype that requires limited development time and is implementable with minimal features while providing a working model with scope for future expansion and improvement. Importantly, given that most of our participants were from two Canadian provinces, it will be important to seek input from a more diverse sample of users during the development and testing phase of the prototype to generate robust data for optimising its utility for diverse older adults with TJR.

## Author Contributions

**Afolasade Fakolade:** conceptualisation, methodology, data curation, funding acquisition, writing–original draft, formal analysis, supervision. **Adriana C. Salvia:** data curation, formal analysis, writing–original draft. **Siona Phadke:** formal analysis, writing–original draft. **Manuela Kunz:** conceptualisation, funding acquisition, writing–review and editing.

## Ethics

Ethical approval was obtained from the Queen's University Health Sciences and Affiliated Teaching Hospitals Research Board (REH‐858‐23). All participants provided informed consent before participating in the study.

## Conflicts of Interest

The authors declare no conflicts of interest.

## Supporting information

Supporting information.

Supporting information.

Supporting information.

## Data Availability

The data generated and analysed during this study to support the findings are included in this published article and its supplementary files.
