# Supplementary material for: An Interactive Vision‐Based 3D Augmented Reality System for In‐Home Physical Rehabilitation: A Qualitative Inquiry to Inform System Development
Source: Health Expect. 2024 Oct 23;27(5):e70020. doi: 10.1111/hex.70020 (PMC11496999; doi:10.1111/hex.70020)
Supplement: Supplementary file 1 — Supporting information. [file HEX-27-e70020-s001.docx]

**Supplementary Material 1**

**Focus Group Guide**

***User requirements for an in-home, augmented reality, physical rehabilitation tool for an aging population***

**Have a copy of the information/consent form; the interview questions and have the slides ready on your computer to share the screen**

**POWERPOINT [There is a slide for each set of questions. Use them to help you manage time per set. It also shows the breakdown of the whole interview to the participants so they know what to expect]**

**Introduction:**

Thank you everyone for being here today.

- Before we begin, I’d like to confirm your consent to participate in this study. [go around and ask each person in the focus group, “e.g., Sarah, do you consent to this study?”]
- At this point, I just need to make sure that everyone has had the chance to complete the online background survey. *[Researcher ensures that everyone has completed these and takes the names of anyone who requires follow-up after the meeting. This step can be done at the same time as verifying consent e.g., “Sarah, do you consent to this study? Did you complete the survey]* We appreciate that you have taken the time to fill these in. This information will help us describe the people who participated in our discussions.
- You have all received the informed consent document in advance of today’s meeting. We are happy to answer any question at all that you might have about the research before we get started, whether it is large or small. If you have questions later on, please feel free to ask.

*[Questions are answered, and participants are invited to ask more questions until there is agreement that there are no further questions.]*

[Once there are no further questions, the researcher continues the introduction.]

Purpose of the study [Tonight/this afternoon/this morning], we will discuss the user requirements for an in-home, augmented reality rehabilitation system.

**Before we begin…**

We want to emphasize that you are free to choose not to answer any question that you don’t wish to. You are also free to sign off the Zoom call at any time [tonight/this afternoon/this morning] should you not feel well or should the topic become too uncomfortable for you to stay. This is a voluntary study, so you are not obligated to participate.

Before we proceed, I would like to go over some logistics to help ensure that the discussion runs smoothly

- Please make sure you are in a quiet location where you will not be disturbed. It is important to make sure that your TV and your radio are off and that you are in a quiet

part of your home. Please mute your microphone when you are not speaking. This prevents undesired audio feedback.

- While we understand that you will realize you have an important point to add to the conversation at certain times, please do your best not to interrupt anyone while they are speaking.
- If we get disconnected and you cannot get back into Zoom, don’t worry, we will follow up with you...
- [Focus group only] I want to emphasize that we are interested in hearing everyone’s opinion … no one opinion is more “right” or more valuable than anyone else’s, so it may help to keep in mind that it is okay if we all don’t agree, but please show respect for others’ opinions.
- If you need to leave the meeting for any reason, please send a private message to me or the co-facilitator *[insert co-facilitator’s name],* via the chat function before you leave. The chat function is a speech balloon located at the bottom of the Zoom screen. Sending me or the co-facilitator *[insert co-facilitator’s name]* a private message reassures us that you are not in any form of distress or health crisis. This will also enable us to follow up with you after the meeting, if necessary.
- Finally, it is important that this focus group discussion be kept confidential. We all need to agree that we will not tell others what was said here to protect the confidentiality of fellow participants. Ask, “Do you all agree to keep today’s discussion confidential?

*[Interviewer scans the Zoom room for verbal and non-verbal signs of agreement.]*

Begin recording at this point and let the participants know that you are recording.

**Start Recording (both zoom to computer and backup audio recorder)**

STATE THE DATE OF FOCUS GROUP AND “HARPTA FOCUS GROUP NUMBER’’

Ok, that’s great. Thanks, everyone. We’re about to move on to the questions.

1. To begin with, I would like to know more about your experience performing prescribed exercises at home after your joint replacement surgery.
   1. What did this feel like for you?
   2. What was easy or difficult about performing exercises at home?
   3. How prepared were you to perform the exercises at home?
   4. What areas do you feel more support would have made a difference in your experience and/or outcome?
   5. What kind of support would have made a difference in your experience and/or outcome?
   6. How do you think the use of technology could support your post-surgery exercises?
2. I would like to know more about your experience with technology in general.
   1. What are some of the technologies that you have used to increase your mobility/exercise in the past or continue to use? E.g., exercise videos, Nintendo Wii, video games etc.
   2. What features have you liked the most about this technology?
   3. What features have you liked the least about this technology?
   4. What are some of the positive experiences you have had using technology in the past?
   5. What are some of the negative experiences you have had using technology in the past?
   6. What issues have you encountered while using this technology? E.g., Accessibility, ease of use etc.

***Break: Check in with the group to see if they would like a break at this point to stretch, use the washroom, etc. If they have their video on, they can turn it off. Resume in 5 -10 minutes.***

**Presentation of the data/live feedback to participants**

*In the letter of information that we sent you, we described how the proposed system works. Let’s have a quick review of the process before we move on to the next round of questions. As you can see in this picture, [Researcher shares their Zoom screen to show a photo of the camera that will be used for the study – See Supplementary Material 2]. We will use this 3D camera to capture information about your body parts, posture, and movements during exercise. The camera connects to your TV where you will be able to see exercise programs. The information will be processed in real-time through the system to enable immediate feedback on your performance and activities during the exercise sessions.*

1. What is your first impression of this tool?

Probe

- What are some of the advantages you think this tool provides?
- What are some of the drawbacks of using this tool?
- Would you consider using this AR system? Why/why not?

*Next, I am going to ask you to imagine an ideal system that would support and guide your movements while performing exercises, what would it look like?*

1. What would be the best way of presenting the exercise guides on the TV screen for you to follow? *[Researcher shares slides with information about different types of data presentation].*
   - E.g., voice, video, 2D images, avatars, etc.
2. Would you prefer a self-guided experience or having real-time feedback?
   - For real-time feedback, what would be the best way to provide the feedback? E.g., voice, video, 2D image, avatars, etc.
   - When would you prefer to receive feedback on your performance – immediately or after the exercise is completed?
3. How would you prefer to navigate the AR system?
   - E.g., voice, through visual cues, by clicking the remote, etc.
   - Do you use any assistive devices that you would like to be connected to the TV?

Next, I'd like to know what would motivate you to engage in an exercise program using this system.

1. If you think about using this tool to increase your activity level, what do you think will help you follow through with your exercises?
2. What factors will hold you back?
3. Thinking about the proposed tool, what type of strategies would help you stick to the program?

Probe

- Tracking and showing improvement over time,
- Showing the timeline of exercises
- Sharing progress with others? Whom would you like to share your progress with? E.g., other participants, close friends, family members, healthcare providers, etc.
- Make it fun? What would make this activity more fun for you? E.g., your choice of the virtual environment - in a park, on a beach, etc.

OK. I see that our time is almost at an end [tonight/this afternoon/this morning]. Just before we wrap up**, is there anything that we have missed or that you have not had a chance to say on this topic?**

*[Researcher facilitates comments and then provides a brief summary of the highlights of the discussion until the end of the allotted time.]*

We would like to thank you again for taking the time out of your busy schedules to share your thoughts and feelings with us about this topic. We look forward to keeping those of you interested up to date on the findings from the focus groups. Please don’t hesitate to let us know if you have any questions or concerns at all, either by phone or email.

We will remain in the Zoom room for a short time afterwards. Please feel free to speak with us afterwards if you have any comments or questions, too.

You will receive your online gift card in the next couple of days.

Thank you for your time.

**Individual Interview Guide**

***User requirements for an in-home, augmented reality, physical rehabilitation tool for an aging population***

**Have a copy of the information/consent form; the interview questions and have the slides ready on your computer to share the screen**

**POWERPOINT [There is a slide for each set of questions. Use them to help you manage time per set. It also shows the breakdown of the whole interview to the participants so they know what to expect]**

**Introduction:**

Thank you, [name of participant], for being here today.

- Before we begin, I’d like to confirm your consent to participate in this study.
- At this point, I just need to make sure that you have had the chance to complete the online background survey. *[Researcher ensures that the participant has completed the survey and notes whether a follow-up is required after the meeting].*
- I appreciate your taking the time to fill out the survey. This information will help us describe the people who participated in our discussions.
- You received the informed consent document in advance of today’s meeting. I am happy to answer any question at all that you might have about the research before we get started, whether it is large or small. If you have questions later on, please feel free to ask.

*[Questions are answered, and the participant is invited to ask more questions until there is agreement that there are no further questions.]*

[Once there are no further questions, the researcher continues the introduction.]

Purpose of the study [Tonight/this afternoon/this morning], we will discuss the user requirements for an in-home, augmented reality rehabilitation system.

**Before we begin…**

I want to emphasize that you are free to choose not to answer any question that you don’t wish to. You are also free to sign off the Zoom call at any time [tonight/this afternoon/this morning] should you not feel well or should the topic become too uncomfortable for you to stay. This is a voluntary study, so you are not obligated to participate.

Before we proceed, I would like to go over some logistics to help ensure that the discussion runs smoothly

- Please make sure you are in a quiet location where you will not be disturbed. It is important to make sure that your TV and your radio are off and that you are in a quiet

part of your home. Please mute your microphone when you are not speaking. This prevents undesired audio feedback.

- If we get disconnected and you cannot get back into Zoom, don’t worry, I will follow up with you...
- If you need to leave the meeting for any reason, please let me know before you leave. Letting me know you have to leave helps me to verify that you are not in any form of distress or health crisis. This will also enable me to follow up with you after the meeting, if necessary.

Begin recording at this point and let the participant know that you are recording.

**Start Recording (both zoom to computer and backup audio recorder)**

STATE THE DATE OF THE INTERVIEW AND “HARPTA INTERVIEW NUMBER’’

Ok, that’s great. Thanks, [name of participant]. We’re about to move on to the questions.

1. To begin with, I would like to know more about your experience performing prescribed exercises at home after your joint replacement surgery.
   1. What did this feel like for you?
   2. What was easy or difficult about performing exercises at home?
   3. How prepared were you to perform the exercises at home?
   4. What areas do you feel more support would have made a difference in your experience and/or outcome?
   5. What kind of support would have made a difference in your experience and/or outcome?
   6. How do you think the use of technology could support your post-surgery exercises?
2. I would like to know more about your experience with technology in general.
   1. What are some of the technologies that you have used to increase your mobility/exercise in the past or continue to use? E.g., exercise videos, Nintendo Wii, video games etc.
   2. What features have you liked the most about this technology?
   3. What features have you liked the least about this technology?
   4. What are some of the positive experiences you have had using technology in the past?
   5. What are some of the negative experiences you have had using technology in the past?
   6. What issues have you encountered while using this technology? E.g., Accessibility, ease of use etc.

***Break: Check in with the participant to see if they would like a break at this point to stretch, use the washroom, etc. If they have their video on, they can turn it off. Resume in 5 -10 minutes.***

**Presentation of the data/live feedback to participants**

*In the letter of information that we sent you, we described how the proposed system works. Let’s have a quick review of the process before we move on to the next round of questions. As you can see in this picture, [Researcher shares their Zoom screen to show a photo of the camera that will be used for the study – See Supplementary Material 2]. We will use this 3D camera to capture information about your body parts, posture, and movements during exercise. The camera connects to your TV where you will be able to see exercise programs. The information will be processed in real-time through the system to enable immediate feedback on your performance and activities during the exercise sessions.*

1. What is your first impression of this tool?

Probe

- What are some of the advantages you think this tool provides?
- What are some of the drawbacks of using this tool?
- Would you consider using this AR system? Why/why not?

*Next, I am going to ask you to imagine an ideal system that would support and guide your movements while performing exercises, what would it look like?*

1. What would be the best way of presenting the exercise guides on the TV screen for you to follow? *[Researcher shares slides with information about different types of data presentation]­.*
   - E.g., voice, video, 2D images, avatars, etc.
2. Would you prefer a self-guided experience or having real-time feedback?
   - For real-time feedback, what would be the best way to provide the feedback? E.g., voice, video, 2D image, avatars, etc.
   - When would you prefer to receive feedback on your performance – immediately or after the exercise is completed?
3. How would you prefer to navigate the AR system?
   - E.g., voice, through visual cues, by clicking the remote, etc.
   - Do you use any assistive devices that you would like to be connected to the TV?

Next, I'd like to know what would motivate you to engage in an exercise program using this system.

1. If you think about using this tool to increase your activity level, what do you think will help you follow through with your exercises?
2. What factors will hold you back?
3. Thinking about the proposed tool, what type of strategies would help you stick to the program?

Probe

- Tracking and showing improvement over time,
- Showing the timeline of exercises
- Sharing progress with others? Whom would you like to share your progress with? E.g., other participants, close friends, family members, healthcare providers, etc.
- Make it fun? What would make this activity more fun for you? E.g., your choice of the virtual environment - in a park, on a beach, etc.

OK. I see that our time is almost at an end [tonight/this afternoon/this morning]. Just before we wrap up**, is there anything that I have missed or that you have not had a chance to say on this topic?**

*[Researcher facilitates comments and then provides a brief summary of the highlights of the discussion until the end of the allotted time.]*

I would like to thank you again for taking the time out of your busy schedules to share your thoughts and feelings with us about this topic. I look forward to keeping you up to date on the study's findings [if interested]. Please don’t hesitate to let me know if you have any questions or concerns at all, either by phone or email.

I will remain in the Zoom room for a short time afterwards. Please feel free to speak with me afterward if you have any comments or questions.

You will receive your online gift card in the next couple of days.

Thank you for your time.
