# Supplementary material for: An Interactive Vision‐Based 3D Augmented Reality System for In‐Home Physical Rehabilitation: A Qualitative Inquiry to Inform System Development
Source: Health Expect. 2024 Oct 23;27(5):e70020. doi: 10.1111/hex.70020 (PMC11496999; doi:10.1111/hex.70020)
Supplement: Supplementary file 2 — Supporting information. [file HEX-27-e70020-s002.docx]

Supplementary Material 2

Welcome to the Focus Group/Interview

for the study:

User requirements for an in-home, augmented reality, physical rehabilitation tool for an aging population (HARPTA)


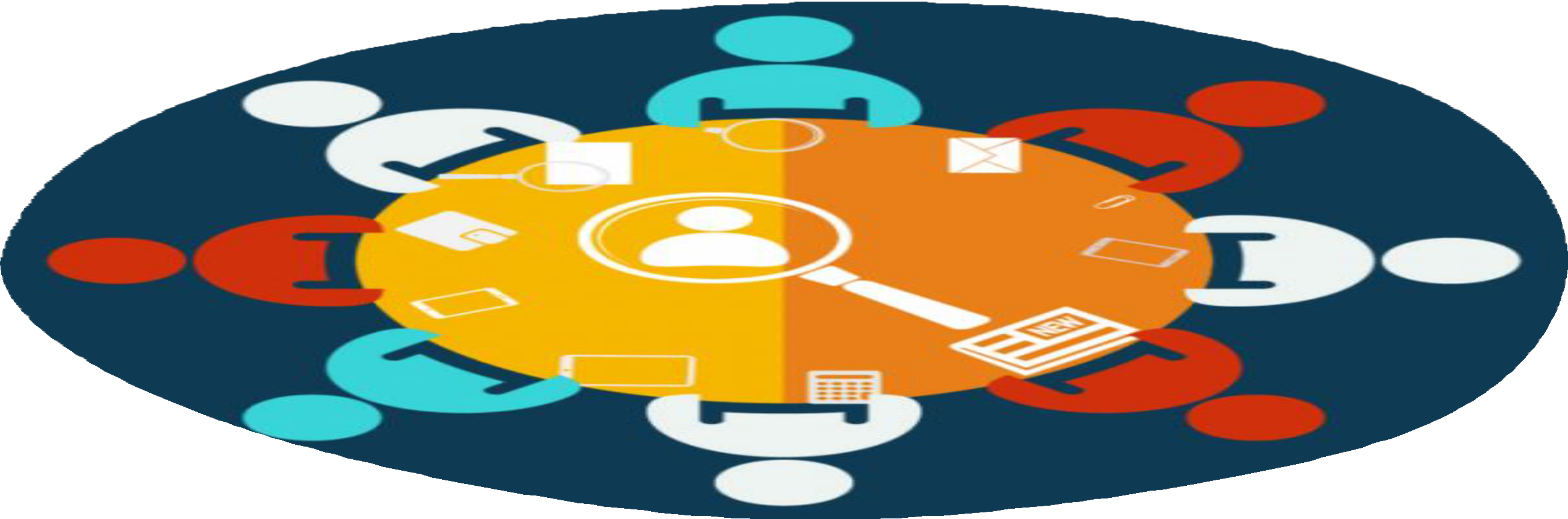


[This Photo](https://academy.hsoub.com/design/user-experience/%D8%A3%D9%81%D8%B6%D9%84-%D8%AE%D9%85%D8%B3-%D8%B7%D8%B1%D9%82-%D8%A7%D8%AE%D8%AA%D8%A8%D8%A7%D8%B1-%D9%84%D9%84%D9%85%D8%B3%D8%AA%D8%AE%D8%AF%D9%85-r385/) by Unknown author is licensed under [CC BY-SA-NC](https://creativecommons.org/licenses/by-nc-sa/3.0/).

- **Verifying consent and survey completion**
- **Study questions**
- **Voluntary participation**
- **Technical issues**
- **Respectful dialogue**


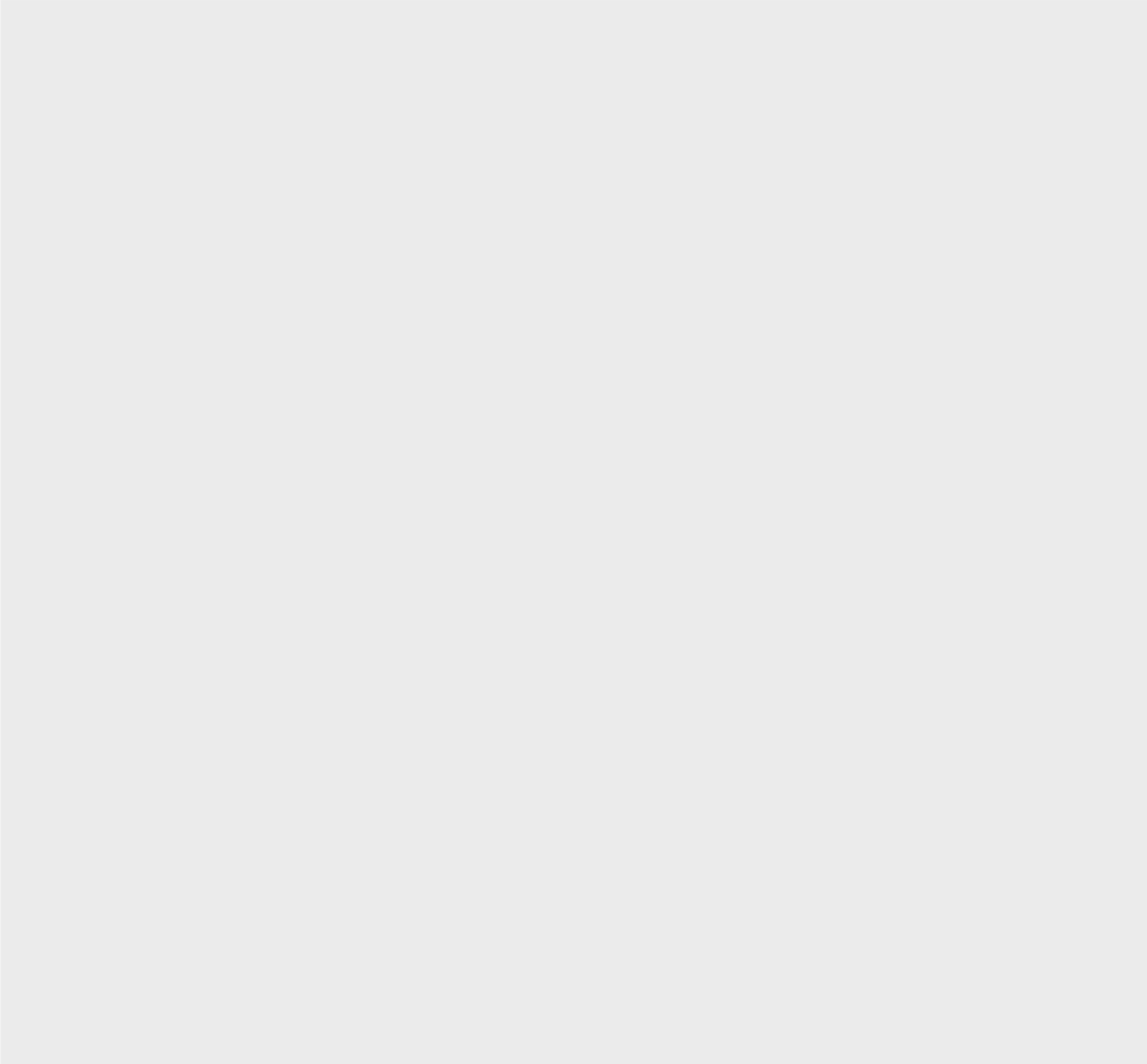


Table of Contents

1. **Experiences with Post-Operative Exercises**
2. **Experience with technology in general**
3. ***5-minute Break***
4. **Example images and feedback**
5. **Questions/comments**

Experience with Post-Operation Exercises


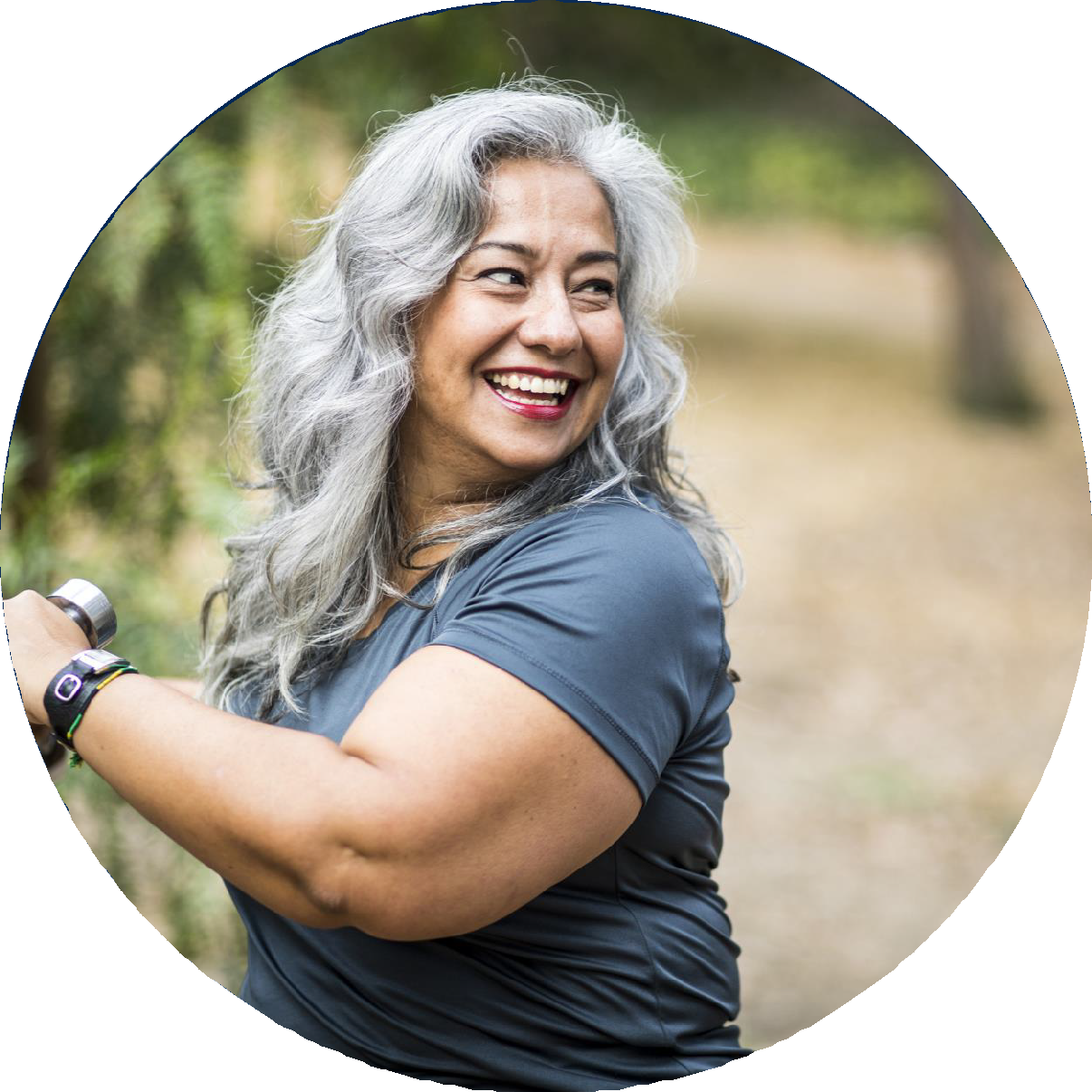

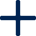

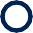

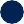


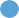

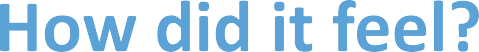

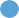

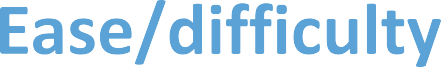

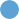

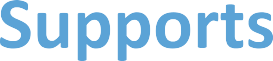

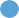

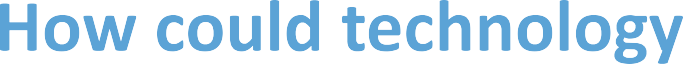

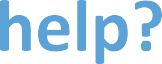


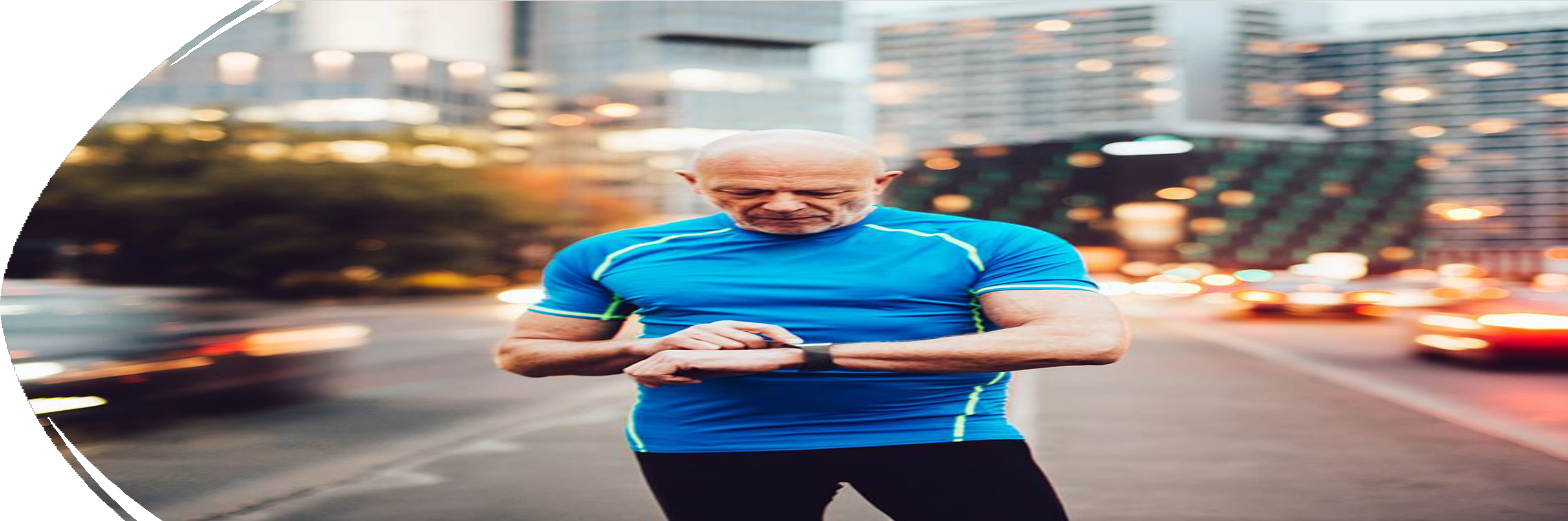


Experience with Technology

- - **Technologies you have used for mobility/exercise**
  - **Features you like/dislike**
  - **Positive/negative experiences**
  - **Issues you have encountered**

Short Break (5 Minutes)


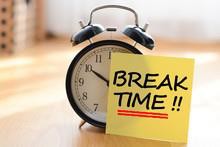


Integrated Camera

- -
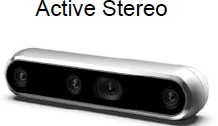
We will use a commercial 3D camera, such as the Intel RealSense D455, to capture information about parts of your body, posture, and movements during exercise. The camera connects to your TV where you

will be able to see exercise programs.

- - The information will be processed in real time through the system to enable immediate feedback on your performance

Intel RealSense^TM^ Depth Camera D455

and activities during the exercise session.
