# Supplementary material for: An Interactive Vision‐Based 3D Augmented Reality System for In‐Home Physical Rehabilitation: A Qualitative Inquiry to Inform System Development
Source: Health Expect. 2024 Oct 23;27(5):e70020. doi: 10.1111/hex.70020 (PMC11496999; doi:10.1111/hex.70020)
Supplement: Supplementary file 3 — Supporting information. [file HEX-27-e70020-s003.docx]

**Supplementary Material 3**

**Coding Framework**

| Codes | Conceptual descriptions |
| --- | --- |
| Standardized Information | Statements about the absence or need for information to prepare all patients completing TJR adequately. |
| Self-motivation | Statements about motivation to complete recommended exercises and achieve successful healing due to their own interest. |
| PT/Other HCP support | Statements about experiences with the physiotherapist and other health care practitioners, including availability to guide and provide feedback on home exercise programming |
| Accessing support services | Statements about challenges accessing external supports to assist during their healing journey. |
| Post-op Complications | Statements about challenges identifying deviations from the normal recovery and indicators of complications |
| ARS Information Display and Feedback | Statements about how visual and sensory information and feedback should be conveyed in the ARS, including sensors to track and guide post-operative exercise |
| Customization and Control | Statements that reflect potential ARS options to facilitate choice and control for customization. |
| Interacting with the ARS | Statements about options for activating and interacting with the ARS e.g., through voice or remote control |
| Technology Proficiency | Statements about previous use of technology in daily life, including skills and training needed to navigate the ARS environment successfully. |
| System Compatibility | Statements about how the ARS will communicate with existing technology used in daily life |
| Social Connection | Statements about the potential for using the ARS for social connections |
| Cost and Accessibility | Statements about cost considerations for accessing and using the ARS |
| Privacy Concerns | Statements reflecting concerns about maintaining privacy and confidentiality while using ARS. |
| Spatial Considerations | Statements about the physical space requirements needed to accommodate the ARS and supporting hardware. |
